# Crassulacean Acid Metabolism Abiotic Stress-Responsive Transcription Factors: a Potential Genetic Engineering Approach for Improving Crop Tolerance to Abiotic Stress

**DOI:** 10.3389/fpls.2019.00129

**Published:** 2019-02-22

**Authors:** Atia B. Amin, Kumudu N. Rathnayake, Won C. Yim, Travis M. Garcia, Beate Wone, John C. Cushman, Bernard W. M. Wone

**Affiliations:** ^1^Department of Biology, University of South Dakota, Vermillion, SD, United States; ^2^Department of Biochemistry and Molecular Biology, University of Nevada, Reno, Reno, NV, United States

**Keywords:** abiotic stress response, crassulacean acid metabolism, drought tolerance, extremophytes, genetic engineering, transcription factor

## Abstract

This perspective paper explores the utilization of abiotic stress-responsive transcription factors (TFs) from crassulacean acid metabolism (CAM) plants to improve abiotic stress tolerance in crop plants. CAM is a specialized type of photosynthetic adaptation that enhances water-use efficiency (WUE) by shifting CO_2_ uptake to all or part of the nighttime when evaporative water losses are minimal. Recent studies have shown that TF-based genetic engineering could be a useful approach for improving plant abiotic stress tolerance because of the role of TFs as master regulators of clusters of stress-responsive genes. Here, we explore the use of abiotic stress-responsive TFs from CAM plants to improve abiotic stress tolerance and WUE in crops by controlling the expression of gene cohorts that mediate drought-responsive adaptations. Recent research has revealed several TF families including *AP2/ERF*, *MYB, WRKY, NAC*, *NF-Y*, and *bZIP* that might regulate water-deficit stress responses and CAM in the inducible CAM plant *Mesembryanthemum crystallinum* under water-deficit stress-induced CAM and in the obligate CAM plant *Kalanchoe fedtschenkoi*. Overexpression of genes from these families in *Arabidopsis thaliana* can improve abiotic stress tolerance in *A. thaliana* in some instances. Therefore, we propose that TF-based genetic engineering with a small number of CAM abiotic stress-responsive TFs will be a promising strategy for improving abiotic stress tolerance and WUE in crop plants in a projected hotter and drier landscape in the 21st-century and beyond.

## Introduction

Formidable challenges facing humankind include a burgeoning global human population (Godfray et al., 2010; Gerland et al., 2014) and the increasing frequency and intensity of droughts related to global warming (Cook et al., 2014; Singh et al., 2015). In addition, abiotic stresses including high salinity, temperature extremes, increased UV radiation, heavy metals, and high light intensities are and will continue to be major constraints for global crop production and food security (Lesk et al., 2016). Among these abiotic stresses, drought is of major concern as it has dire effects on crop productivity (Fahad et al., 2017), plant growth, and development (Yordanov et al., 2000). By the end of the 21st century, rapid changes in the global climate will likely increase the frequencies of drought by more than 20% compared to current rates (Lobell et al., 2011; Cook et al., 2014). Indeed, Daryanto et al. (2016) showed that an approximately 40% decrease in water availability can decrease wheat (*Triticum aestivum* L.) and maize (*Zea mays* L.) yields by 20.6% and 39.3%, respectively. High salinity, another abiotic stress that is harmful to crop production, affects 20% of total cultivated and 33% of irrigated agricultural lands worldwide (Shrivastava and Kumar, 2015). Similarly, low temperatures and heatwaves cause significant reduction in crop yields across the world (Sanghera et al., 2011; Challinor et al., 2014; Hatfield and Prueger, 2015). Thus, novel approaches to mitigate the negative impacts of abiotic stresses on crop yields must be explored and developed to avoid socio-economic collapse due to climate change.

Approaches to enhance sustainable bioenergy production by engineering the CAM pathway into C_3_ crops to enhance their water-use efficiency (WUE) on marginal lands are already underway (Borland et al., 2014, 2015; Yang et al., 2015; Liu et al., 2018). Another approach to enhance abiotic stress tolerance is to modulate the expression of transcription factors (TFs) or the functions of abiotic stress-adaptive genes that might already be present, but that are not normally expressed in unstressed or C_3_ plants (Hussain et al., 2011; Rabara et al., 2014; Joshi et al., 2016; Wang et al., 2016; Bechtold, 2018). This approach would involve bioengineering a small number of regulatory genes with potentially global effects made possible by the role of TFs in gene regulation (Rabara et al., 2014; Joshi et al., 2016).

Transcription factor-based genetic engineering could direct such regulatory TFs to modulate a large number of downstream abiotic stress-responsive genes (Rabara et al., 2014). Stress tolerance in plants is generally under polygenic control (Tran et al., 2010) and some of the genes regulating stress-tolerance responses happen to code for TFs (Villalobos et al., 2004; Qiu and Yu, 2009; Zhang et al., 2011; Yang et al., 2012; Cai et al., 2014; Chen et al., 2014; Swain et al., 2017). Therefore, TFs might be ideal candidate regulators for improving abiotic stress tolerance in crop plants. To date, TF-based genetic engineering has mainly repurposed TFs from *Arabidopsis thaliana*, *Glycine max*, *Oryza sativa*, and *T. aestivum* (Rabara et al., 2014; Joshi et al., 2016; Wang et al., 2016). As far as we know, no reports have analyzed the effects of overexpressing TFs from crassulacean acid metabolism (CAM) plants which have greater abiotic stress tolerances than mesophytes. Most CAM plants are naturally adapted to low-water environments and many other abiotic stresses compared to the agronomically important C_3_ plants (Borland et al., 2009). Here, we consider using a “next-generation TF-based” approach to exploit abiotic stress-responsive TFs from CAM plants to improve abiotic stress tolerance in crop plants. The current technologies for TF-based approaches to improve plant abiotic stress tolerance have been extensively discussed and reviewed by Rabara et al. (2014), Joshi et al. (2016), and Wang et al. (2016), and those details are therefore only briefly summarized below.

## Transcription Factor-Based Approach

Plants are sessile organisms that exhibit various biochemical, physiological, and molecular adaptations to extreme environments (Joshi et al., 2016). For instance, water-deficit stress activates the expression of stress-responsive genes encoding enzymes that synthesize compatible protective sugars, antioxidants, and proteins, including heat shock proteins and some classes of late embryogenesis abundant (LEA) proteins (Tran et al., 2010; Joshi et al., 2016; Wang et al., 2016). In addition to the stress-induced upregulation of the above proteins, the expression of various regulatory proteins including TFs, protein kinases, and protein phosphatases is also activated (Wang et al., 2016).

Transcription factors are master regulators of many cellular processes and can also interact with other transcriptional regulators (Joshi et al., 2016). Importantly, they play a pivotal role in different abiotic stress responses by binding to the upstream *cis*-regions of promoters in many stress-responsive genes (Yamaguchi-Shinozaki and Shinozaki, 2006). Many studies have been conducted to identify and characterize families of TFs including *AP2/ERFBP*, *MYB*, *WRKY*, *NAC*, *NF-Y*, and *bZIP* that are involved in abiotic stress responses (Umezawa et al., 2006; Golldack et al., 2011; Leyva-González et al., 2012; Wang et al., 2016; Swain et al., 2017; Zanetti et al., 2017). Several TFs have already been overexpressed in crop plants and *A. thaliana* to improve abiotic stress tolerance (Qiu and Yu, 2009; Zhang et al., 2011; Yang et al., 2012; Cai et al., 2014; Chen et al., 2014). For example, the NAC family is one of the largest TF families in plants and is involved not only in plant growth and development, but also in transcriptional reprogramming associated with plant stress responses (Tran et al., 2010; Nakashima et al., 2012). Mao et al. (2012) reported that overexpression of the *TaNAC2* gene from wheat can enhance tolerance to drought, salt, and freezing stresses in *A. thaliana*. In addition, functional characterization of the *NAC045* (Zheng et al., 2009) and *SNAC1* genes from *O. sativa* enhanced drought and salt tolerance in rice (Hu et al., 2006). Furthermore, overexpression of either *GmMYB76* or *GmMYB177* from soybean significantly enhanced salt and freezing tolerance in *A. thaliana* (Liao et al., 2008).

A relatively less explored yet high-potential approach is to discover novel abiotic stress-adaptive regulatory genes in extremophytes (i.e., CAM xerophytes and halophytes, desiccation-tolerant plants, or resurrection plants) to use for bioengineering abiotic stress tolerance in crop plants (Inan et al., 2004; Shi et al., 2013; Joshi et al., 2016; Bechtold, 2018). For example, the overexpression of the TF *CpMYB10* from the resurrection plant *Craterostigma plantagineum* in *A. thaliana* led to desiccation and salt tolerance in transgenic lines (Villalobos et al., 2004).

## Crassulacean Acid Metabolism and Abiotic Stress Tolerance

CAM plants have evolved a specialized type of photosynthetic adaptation that allows them to live under conditions of severe water deficit and in semi-arid and arid regions of the world including deserts. These plants have shifted all or part of their primary CO_2_ uptake and fixation to the nighttime, when evaporative water losses are minimal, and perform C_3_ carboxylation reactions when stomata are closed during the daytime. This temporal separation of carbon fixation leads to the formation of the four-carbon organic acid malate, which is stored in the vacuole during the night and subsequently undergoes decarboxylation to release CO_2_ for re-fixation during the day to produce carbohydrates (Borland et al., 2009). Because of this temporal separation of carbon fixation and inverted stomatal behavior, CAM plants can reduce water loss due to transpiration. These characteristics also allow CAM plants to fix net CO_2_ 15% more efficiently than C_3_ plants (Nobel, 1991) resulting in increased biomass of CAM plants while using less water than C_3_ plants. Additionally, CAM plants can produce similar amounts of biomass using 80% less water in comparison to C_3_ plants (Nobel, 1996; Borland et al., 2009). Thus, CAM plants have between 3- and 6-fold higher WUE than C_4_ and C_3_ plants, respectively (Garcia et al., 2014; Yang et al., 2015).

In addition to their higher WUE and associated drought tolerance (Yang et al., 2017), CAM plants can tolerate high temperature up to 70°C, whereas C_3_ plants can tolerate only 50–55°C (Borland et al., 2009). CAM halophytes can also adapt to high salinity, as during the induction of CAM by salt stress in *Mesembryanthemum crystallinum* (Winter and Holtum, 2014). Moreover, CAM plants can better tolerate higher light intensities (>1000 μmol m^-2^ s^-1^) and UV-B irradiation levels than can agronomically important C_3_ plants (Borland et al., 2009). Furthermore, CAM plants can increase daily net CO_2_ uptake under increased atmospheric CO_2_ concentrations, which might be advantageous in global climate change scenarios (Nobel, 1996). Some CAM plants such as *Agave salmiana, Opuntia ficus-indica*, and *Stenocereus queretaroensis* can also survive in subzero temperatures, and *Agave utahensis* Engelm. can tolerate temperatures as low as -18°C (Nobel, 1996).

The abiotic stress-adaptive characteristics of CAM plants will be particularly beneficial for adapting to the consequences of anthropogenic climate change, such as droughts and heatwaves, high soil salinity, temperature extremes, and high light or UV-B irradiation. Many of the stress-adaptive responses involve abscisic acid (ABA)-dependent and -independent response pathways (Song et al., 2016). ABA-dependent and independent signaling events likely participate in the stress-activation of CAM in *M. crystallinum* (Chu et al., 1990; Taybi and Cushman, 1999, 2002; Abdin et al., 2002; Cushman and Borland, 2002), suggesting that the CAM pathway likely has evolved in response to abiotic stress (Reyes-García and Andrade, 2009; Heyduk et al., 2018; Yin et al., 2018).

Crassulacean acid metabolism is thought to have evolved independently multiple times from ancestral C_3_ plants (Silvera et al., 2010) because no unique metabolic pathways are required, although some CAM-specific variant enzymes apparently evolved in some instances (Ermolova et al., 2003; Gehrig et al., 2005; Vaasen et al., 2006). However, temporal changes in gene expression of the CAM enzyme variants likely occurred because of alterations in their regulation compared to their orthologs in C_3_ plants (Hermans and Westhoff, 1990; Lepiniec et al., 1993; Cushman et al., 2008; Heyduk et al., 2018; Yin et al., 2018). Furthermore, the ABA-dependent stress response pathway is involved in CAM activation not only in *M. crystallinum* (Taybi and Cushman, 2002), but also in other CAM species (Taybi et al., 1995; Rodrigues et al., 2016; Yin et al., 2018). Although CAM is found in over 36 families of vascular plants (Silvera et al., 2010), we rely on only a few major CAM species such as pineapple (*Ananas comosus*), *Agave*, and *Opuntia* as agricultural crops to provide food, forage, fiber, and biofuels (Cushman et al., 2015). Well characterized CAM model species also provide abundant resources for the identification and selection of candidate TFs involved in abiotic stress adaptations (Hartwell et al., 2016). Hence, identification of candidate CAM pathway regulators (i.e., TFs) that are expressed or activated under water-deficit stress or CAM should be prioritized to exploit the molecular and regulatory machinery of abiotic stress adaptation in CAM plants as a vital resource for applications in C_3_ crop species (Yang et al., 2015; Fernie, 2016; Yin et al., 2018).

## Cam Abiotic Stress-Responsive TF-Based Approach

Bioengineering a TF that can confer desirable traits such as increased drought tolerance (Villalobos et al., 2004) or increased biomass (Lim et al., 2018) into C_3_ *A. thaliana* will be crucial as a proof of concept for the CAM abiotic stress-responsive TF-based approach to increase abiotic stress tolerance in C_3_ plants. Fortunately, genetic resources (i.e., genome and transcriptome sequences) for CAM plants are now available for *Agave* (Abraham et al., 2016), *Kalanchoe* spp. (Yang et al., 2017), pineapple (Ming et al., 2015, 2016; Wai et al., 2017), and *M. crystallinum* (Chiang et al., 2016). Although genetic resources for CAM plants are becoming readily available, the underlying regulatory basis of CAM is still not completely understood. Many TFs of unknown function have been identified during recent genome and transcriptome sequencing efforts; thus, there are now many opportunities to analyze the functions of TFs involved in water-deficit-stress response or CAM function and to exploit the potential of bioengineering using CAM plant TFs to improve abiotic stress tolerance in crop plants. Indeed, candidate CAM TFs involved in C_3_ to CAM transition in obligate CAM species of *Agave* (Heyduk et al., 2018; Huang et al., 2018; Yin et al., 2018), *Kanlanchoe* (Moseley et al., 2018), and *Manfreda* (Heyduk et al., 2018), and weak CAM species of *Polianthes* and *Beschorneria* (Heyduk et al., 2018), or the induction of CAM in *Tralinum triangulare* (Brilhaus et al., 2016) have been identified. Not surprisingly, a number of these candidate TFs are involved in the ABA stress responsive pathway (Heyduk et al., 2018; Yin et al., 2018). More importantly though, many of these candidate TFs are involved in the rewiring of the phase shift from C_3_ to CAM transition in the evolution of CAM photosynthesis (Heyduk et al., 2018; Moseley et al., 2018; Yin et al., 2018). Although it would be interesting to attempt to reprogram a C_3_ plant such that it becomes CAM performing, we are not suggesting to shift gene expression patterns of CAM pathway genes that might be present in extant C_3_ plants, or to regulate the engineered CAM pathway in C_3_ plants (Yang et al., 2015; Fernie, 2016; Heyduk et al., 2018; Yin et al., 2018), but rather identify and exploit the TFs involved in abiotic stress responses from obligate and inducible CAM plants to modulate the expression of appropriate genes in C_3_ plants to improve their abiotic stress tolerance.

Eight most abundant candidate TFs under water-deficit stress diel and zeitgeber time have been identified that might regulate the CAM state, water-deficit stress response, or both in *M. crystallinum* (Garcia et al., 2014; Cushman, unpubl. data; Table 1). *Mesembryanthemum crystallinum* switches from C_3_ to CAM when salt or water-deficit stressed (Cushman, 2001; Cushman and Borland, 2002; Taybi and Cushman, 2002). *Kalanchoe fedtschenkoi* orthologs of these top candidate TFs were also highly expressed during CAM induction in older leaf pairs of *K. fedtschenkoi* plants (Garcia et al., 2014; Cushman, unpubl. data). It is known that young leaves are C_3_ performing, whereas mature leaves are CAM performing in *K. fedtschenkoi* (Cushman, 2001). These TFs share a base mean expression level of >100 FPKM and at least two-fold induction during a transition from C_3_ to CAM or imposition of water-deficit stress in *K. fedtschenkoi* and *M. crystallinum*, respectively (Garcia et al., 2014; Cushman, unpubl. data). These candidate CAM TFs also belong to the families of TFs reported to be involved in abiotic stress responses (Rabara et al., 2014; Joshi et al., 2016; Roy, 2016; Wang et al., 2016). Orthologs of these candidate CAM TFs in *A. thaliana* also have several reported functions in plant development and abiotic stress tolerance (Zheng et al., 2009; Zhang et al., 2011; Yang et al., 2012; Chen et al., 2014; Swain et al., 2017; Zanetti et al., 2017). Intriguingly, recent functional characterization of two putative CAM regulators of water-deficit stress response or CAM activation *via* overexpression in *A. thaliana* strongly suggest that CAM TFs have high potential to increase tolerance to drought and other abiotic stresses in C_3_ plants.

**Table 1:** List of the top eight candidate transcription factors (TFs) from the inducible CAM plant *Mesembryanthemum crystallinum* and top eight candidate TFs from the obligate CAM plant *Kalanchoe fedtschenkoi* hypothesized to regulate the CAM state or water-deficit stress responses in CAM plants and their corresponding orthologs in *Arabidopsis thaliana*.

TF Name	TF Family	*A. thaliana* Locus ID	Functional annotation of *A. thaliana* ortholog at TAIR
*McERF74*	AP2/ERF/CRF	AT1G53910	Detection of hypoxia, ethylene-activated signaling pathway, regulation of root development, response to hypoxia
*McNAC29*	NAC	AT1G69490	Embryo development ending in seed dormancy, flower development, fruit ripening, leaf senescence, multicellular organism development, multidimensional cell growth, regulation of transcription
*McBLH1*	HB/Homeodomain	AT2G35940	Polar nuclei fusion, response to abscisic acid, response to continuous far red-light stimulus by the high-irradiance response system, response to symbiotic fungus
*McbZIP2*	bZIP	AT2G18160	Positive regulation of transcription
*McAGL8*	MADS/AGAMOUS -LIKE 8	AT5G60910	Cell differentiation, developmental growth involved in morphogenesis, positive regulation of flower development, fruit development, maintenance of inflorescence meristem identity
*McAP2-12*	AP2/ERF	AT1G53910	Detection of hypoxia, ethylene-activated signaling pathway, regulation of root development, response to hypoxia
*McbZIP44*	bZIP	AT1G75390	Positive regulation of transcription, seed germination
*McHB7*	HB/Homeobox	AT2G46680	Abscisic acid-activated signaling pathway, positive regulation of transcription, response to water deprivation
*KfMYB59*	MYB	AT5G59780	Cell differentiation, response to cadmium ions, response to ethylene, response to gibberellin, response to NaCl
*KfLHY1*	Homeodomain	AT1G01060	Circadian rhythm, long-day photoperiodism, flowering, response to abscisic acid, response to auxin, response to NaCl
*KfBZIP29*	bZIP	AT4G38900	Regulation of transcription, reproductive shoot system and development
*KfNF-YB3*	NF-Ys	AT4G14540	Regulation of transcription, protein heterodimerization
*KfNAC83*	NAC	AT5G13180	Lignin biosynthetic process, plant-type secondary cell wall biogenesis, fruit dehiscence
*KfAP2*	AP2/ERF/CRF	AT4G11140	Cotyledon development cytokinin-activated signaling pathway, ethylene-activated signaling pathway, leaf development
*KfCOL3* (010148t1)	Zinc Finger CONSTANS-like 4	AT5G24930	Red light signaling pathway, regulator of flower development, regulation of photomorphogenesis
*KfCOL5* (00914t1)	Zinc Finger CONSTANS-like 5	AT5G57660	Regulation of flower development, regulation of transcription, response to light stimulus


*We are only reporting functions related to transcriptional activation and abiotic stress for the A. thaliana ortholog from the Arabidopsis Information Resource (TAIR) databases (https://www.arabidopsis.org/) in the table.*

One of these candidate CAM TFs is a myeloblastosis (MYB59, closest ortholog in *Arabidopsis*) TF whose transcripts are 20-fold more abundant in CAM-performing older leaf pairs relative to C_3_-performing younger leaves in *K. fedtschenkoi* (Hartwell et al. unpubl. data). Results from four, third-generation (T_3_) transgenic lines carrying the *KlMYB59* indicate increased rosette size and biomass at 4-week-old juvenile stage, and increased shoot length at 8-week-old mature stage in transgenic plants compared to WT (Table 2; Wone et al., unpubl. data; full results being presented in a separate publication). However, transgenic lines show delayed flowering in long-day photoperiod compared to WT plants (16 h light/8 h dark). In addition, these transgenic lines exhibit increased integrated WUE compared to WT plants. Furthermore, transgenic lines have longer primary roots despite exposure to 50 μM selenium compared to WT plants. In addition to MYB59, transcripts of the NAC83 TF (closest ortholog in *Arabidopsis*) were also more highly expressed in CAM-performing leaves of *K. laxiflora* and *K. fedtschenkoi* relative to C_3_-performing leaves (Cushman et al., unpubl. data). The function of this *K. fedtschenkoi* NAC83 TF (*KfNAC83*) is not known in CAM- or C_3_-performing CAM plants, but its *A. thaliana* ortholog suggests roles in abiotic stress responses and development (Table 1). Functional characterization of *KfNAC83* shows enhanced water-deficit stress tolerance and increased integrated WUE in four independent transgenic T_3_-generation *A. thaliana* lines compared to WT plants (Table 2; Wone et al., unpubl. data; full results being presented in a separate publication). Furthermore, *KfNAC83*-overexpressing lines show significantly increased rosette size, leaves in the mature rosette, shoot biomass, number of siliques, and lateral roots compared to WT. Interestingly, these transgenic lines also showed tolerance to 150 mM NaCl. Collectively, our characterization results strongly suggest that at least two of these candidate CAM TFs have functions in abiotic stress responses and CAM photosynthesis.

**Table 2:** Results from T_3_ transgenic *A. thaliana* lines overexpressing candidate CAM TFs from the obligate CAM plant *K. fedtschenkoi* hypothesized to regulate water-deficit stress response or CAM activation.

CAM-related transcription factor	Integrated WUE	Drought tolerance	NaCl tolerance	Heavy metal tolerance	Biomass	Timing of bolting	Lateral roots	Root hairs
*KfMYB59*	Enhanced	NC	100 mM NaCl	50 μM Na_2_SeO_4_	Increased	Delayed	NC	Increased
*KfNAC83*	Enhanced	Enhanced	150 mM	TBD	Increased	NC	Increased	Increased


*NC - No change, same as WT; TBD - To be determined.*

## Conclusion

The abiotic stress-adaptive features of CAM plants provide a wealth of genetic resources, specifically TFs, that are now available for functional testing and possible improvement of WUE and abiotic stress responses in C_3_ photosynthesis plants. Our recent findings strongly suggest that a bioengineering approach using CAM abiotic stress-responsive TFs has the potential to increase abiotic stress tolerance in *A. thaliana* and possibly in C_3_ crop plants. Our results indicate that CAM abiotic stress-responsive gene expression can be modulated by the appropriate CAM TFs to generate stress-adaptive phenotypes in *A. thaliana* and likely other C_3_ plants because these CAM abiotic stress-responsive genes are apparently conserved and present in C_3_ plants (Heyduk et al., 2018). Furthermore, although *K*. *fedtschenkoi* is distantly related to *A. thaliana*, transgenic *A. thaliana* lines carrying the obligate CAM plants’ TFs showed favorable features for translational applications. We are optimistic that overexpressing TFs from the inducible CAM halophyte, *M. crystallinum* will have similar favorable responses in *A. thaliana* lines. Co-overexpression of only a small number of obligate and/or inducible CAM plant abiotic stress-responsive TFs with demonstrated abiotic stress-adaptive or -responsive functions would provide a facile approach for bioengineering desirable responses to abiotic stress (Song et al., 2016). Such an approach could open the door to potentially transformative applications to ensure long-term sustainable food, fiber, feed, and fuel production in a projected hotter and drier landscape in the 21st century and beyond.

## Author Contributions

BWMW conceived the CAM abiotic stress-responsive TF-based approach to improve C_3_ crop plant abiotic stress responses. AA, KR, and BWMW wrote the manuscript. AA, KR, and BW conducted the experiments and provided the CAM plant TF overexpression data. WY, TG, and JC provided the CAM plant TF sequences. All authors reviewed the final manuscript.

## Conflict of Interest Statement

The authors declare that the research was conducted in the absence of any commercial or financial relationships that could be construed as a potential conflict of interest.
